# Analysis of the tumor reactivity of autologous TILs and allogeneic γδ T cells via tumor organoid–immune cell coculture

**DOI:** 10.1186/s12967-026-07706-0

**Published:** 2026-04-25

**Authors:** Zijun Su, Haishan Li, Dongdong Zhang, Nanxi Shi, Chanchan Song, Yu Huang, Weili He, Zhinan Yin, Liangping Li

**Affiliations:** 1 School of Medicine, Jinan University, Guangzhou, 510632 China; 2 Guangdong Provincial Key Laboratory of Tumor Interventional Diagnosis and Treatment, Zhuhai People’s Hospital, Zhuhai Institute of Translational Medicine, Zhuhai Medical College of Jinan University, Zhuhai, 519000 China; 3 Clinical Laboratory, Zhongshan Boai Hospital, Zhongshan, Guangdong 528400 China; 4 Department of Thoracic Surgery, The First Affiliated Hospital, Jinan University, Guangzhou, China; 5 The College of Life Science and Technology, Jinan University, Guangzhou, 510632 China; 6 Institute of Clinical Oncology, Research Center of Cancer Diagnosis and Therapy, and Department of Clinical Oncology, The First Affiliated Hospital of Jinan University, Guangzhou, China; 7 The Biomedical Translational Research Institute, Key Laboratory of Viral Pathogenesis & Infection Prevention and Control, School of Medicine, Jinan University, Guangzhou, 510632 China; 8 Department of Breast Surgery, The First Affiliated Hospital of Jinan University, Guangzhou, China; 9 The Affiliated Guangdong Second Provincial General Hospital of Jinan University, Guangzhou, Guangdong 510317 China; 10 State Key Laboratory of Bioactive Molecules and Druggability Assessment, the Biomedical Translational Research Institute, Health Science Center (school of Medicine), Jinan University, Guangzhou, 510632 Guangdong China; 11 Department of Radiotherapy, General Hospital of Southern Theatre Command, Guangzhou, China; 12 Guangzhou City Tianhe District Maternal and Child Health Care Hospital, Guangzhou, China

**Keywords:** γδ T cells, Tumor microenvironment (TME), Immunotherapy, Tumor-infiltrating lymphocytes (TILs), Tumor organoids, Coculture models, Vγ9Vδ2 T cells

## Abstract

**Background:**

The efficacy of conventional αβ T cell-based immunotherapies is often limited by tumor immune evasion. γδ T cells can bypass these limitations through MHC-independent tumor recognition, but their function within the tumor microenvironment (TME) remains poorly characterized due to a lack of relevant preclinical models. This study aims to establish a patient-derived tumor organoid-immune cell coculture system to evaluate γδ T-cell reactivity and cytotoxicity in a human-relevant TME (*n* = 10 PDTO lines; *n* = 3 independent experiments).

**Methods:**

We developed an innovative coculture system—believed to be the first to integrate patient-derived tumor organoids (PDTOs) and autologous tumor-infiltrating lymphocytes (TILs) or healthy donor-derived allogeneic Vγ9Vδ2 T cell. T-cell activation was quantified by comparing CD137 expression on γδ T cells versus CD4^+^ and CD8^+^ T cells via flow cytometry after coculture. The cytotoxicity of Vγ9Vδ2 T cells was evaluated at various effector-to-target (E:T) ratios using a live-cell imaging assay to track BCO infiltration and apoptosis over 24 hours. Secreted effector molecules (IFN-γ, granzyme B, perforin) were measured from coculture supernatants using a cytometric bead array.

**Results:**

Baseline analysis revealed that γδ T cells represent the most reactive subset within expanded TILs, with 12.3% expressing CD137 compared to 3.49% of CD8^+^ T cells (*p* = 0.0118). Notably, autologous BCO–TIL coculture preferentially enhanced γδ T-cell activation, reaching 24.85% CD137^+^—a frequency significantly higher than that of CD8 (9.15%, *p* = 0.0062) and CD4 (9.99%) subsets. The net increase in CD137 positivity for γδ T cells was more than double that of CD8 T cells (12.55% vs. 5.66%, *p* = 0.0467). Allogeneic Vγ9Vδ2 T cells demonstrated significant, dose-dependent cytotoxicity against BCOs (*p* < 0.0001). Maximal cell death (RFU fold-change: 5.2 ± 0.5) was achieved at an effector-to-target (E:T) ratio of 10:1 (*p* < 0.0001). This cytotoxic efficacy was highly correlated with the secretion of lytic effectors, including IFN-γ ( > 1000 pg/mL; *p*< 0.001), granzyme B ( > 2000 pg/mL), and perforin ( > 350 pg/mL).

**Conclusion:**

Our findings demonstrate that 3D autologous coculture leads to the preferential stimulation of γδ T cells, establishing them as a primary reactive subset capable of bypassing MHC-restriction bottlenecks. The potent dismantling of tumor architecture by allogeneic Vγ9Vδ2 T cells, supported by high-resolution kinetic and cytokine data, substantiates their development as “off-the-shelf” therapies. Collectively, this platform provides a standardized, high-fidelity engine for the rapid preclinical assessment of next-generation cellular immunotherapies.

**Supplementary Information:**

The online version contains supplementary material available at 10.1186/s12967-026-07706-0.

## Highlights

A patient-derived breast cancer organoid (BCO)–immune cell coculture system is established to mimic the tumor microenvironment.

Autologous γδ T cells exhibit superior baseline reactivity and achieve preferential tumor-induced activation (24.85% CD137^+^) after BCO coculture.

Allogeneic Vγ9Vδ2 T cells mediate potent, dose-dependent cytotoxicity against BCOs (maximal efficacy at 10:1 E:T ratio).

Cytotoxicity of Vγ9Vδ2 T cells is driven by IFN-γ, granzyme B, and perforin secretion.

The coculture system serves as a preclinical tool for evaluating γδ T cell-based immunotherapies.

**Supplementary Information:**

The online version contains supplementary material available at 10.1186/s12967-026-07706-0.

## Introduction

Cancer remains a leading global health challenge, profoundly impacting social and economic stability worldwide [1, 2]. The genesis of malignancies involves intricate interactions between genetic predispositions and environmental factors, necessitating the continuous advancement of therapeutic strategies [3, 4].

Chemotherapy remains a cornerstone of therapeutic protocols for solid tumors, playing an indispensable role across diverse clinical contexts. Conventional chemotherapeutic agents function primarily by disrupting the highly conserved processes of cell cycle progression and inducing apoptosis in rapidly proliferating cells, thereby exploiting the hyperproliferative phenotype of cancer cells relative to normal somatic cells [5]. However, the clinical utility of these regimens is frequently compromised by limited selectivity, leading to severe systemic toxicity and the development of multidrug resistance (MDR) mechanisms that facilitate tumor survival [4, 6].

A key breakthrough in this area is the development of immune checkpoint inhibitors (ICIs), which block inhibitory pathways such as the PD-1/PD-L1 axis to restore anti-tumor T-cell function, leading to durable clinical responses in cancers such as melanoma and non-small-cell lung cancer [5].

Cancer immunotherapies leverage the immune system’s intrinsic capacity to combat malignancies. However, conventional immunotherapy approaches, which predominantly involve the use of αβ T cells, face significant limitations [7, 8], as αβ T cells recognize tumor-derived peptides through HLA-restricted antigen presentation, a pathway frequently compromised by immune evasion mechanisms such as downregulation of MHC class I molecules, disruption of antigen processing, and immunosuppressive checkpoint signaling within the tumor microenvironment (TME), leading to suboptimal therapeutic responses or even treatment failure in many patients. These impairments directly prevent αβ T cells from efficiently recognizing and eliminating malignant cells [9]. In essence, by disrupting this recognition pathway, tumors can render themselves effectively invisible to the primary effector cells of conventional immunotherapy. Moreover, although immune checkpoint inhibitors (ICIs) and adoptive cell therapies (ACTs), including clinically approved tumor-infiltrating lymphocyte (TIL) therapies (a specific ACT form using immune cells naturally in tumors, which often have inherent reactivity to the patient’s own tumor), have shown therapeutic promise [10–14], their efficacy remains variable and often limited [15, 16]. This is because TIL products frequently contain substantial populations of bystander cells without specific tumor reactivity [17] and because the immunosuppressive TME selectively impairs the effector function of CD8^+^ T cells [18, 19]. Crucially, the use of αβ T cells has been prioritized in the development of ACTs, while the therapeutic potential of unconventional lymphocyte subsets has been largely ignored [20]. This highlights the need to identify other lymphocyte subsets with enhanced antitumor efficacy to overcome the limitations of current αβ T cell-mediated immunotherapies.

γδ T cells represent an unconventional subset of T lymphocytes bridging innate and adaptive immunity. Unlike αβ cells, γδ T cells recognize stress-induced ligands and nonpeptidic antigens, such as phosphoantigens, independently of MHC restriction [20–22]. This distinctive MHC-independent mode of antigen recognition enables γδ T cells to bypass tumor escape strategies that impair αβ T-cell responses, such as loss of HLA class I expression and altered antigen presentation pathways [23]. γδ T cells exhibit potent antitumor activity through direct cytotoxicity, notably granzyme- and perforin-mediated killing, and through immunomodulation, particularly via IFN-γ production [24]. Recent multiomics analyses have suggested that higher intratumoral γδ T-cell abundance is positively correlated with better clinical outcomes in aggressive cancers, including triple-negative breast cancer [25].

Among γδ T-cell subsets, Vγ9Vδ2 T cells, which are prevalent in peripheral blood and are readily expandable from healthy donors on a large scale, present significant translational advantages, particularly for the development of ‘off-the-shelf’ allogeneic therapies, owing to their relatively uniform and conserved T cell receptor (TCR) repertoire across individuals. Importantly, the use of such allogeneic therapies circumvents the need for patient-derived T cells, which are often scarce and functionally impaired by tumor-induced immunosuppression [21]. Early-phase clinical trials have begun to validate this approach; for instance, the foundational work by Xu et al. demonstrated that infusions of allogeneic Vγ9Vδ2 T cells in patients with late-stage lung and liver cancer were remarkably safe, with no graft-versus-host disease observed, and resulted in significantly prolonged overall survival [26]. Building on these promising safety and survival outcomes, a key goal for the field is to further enhance the direct anti-tumor potency of these cells to improve objective response rates in solid tumors [10, 27]. Early-phase clinical trials have validated the safety and preliminary feasibility of Vγ9Vδ2 T cell treatment for multiple tumor types [26, 28]. Although γδ T cells demonstrate substantial therapeutic promise because of their ability to recognize tumor cells independently of MHC molecules [29], the fundamental questions of their functional characteristics and their direct contribution to tumor control as a component of the natural, autologous TIL response remain incompletely understood.

However, the inability of conventional in vitro models to capture the complex spatial organization and cellular diversity inherent to the TME poses a significant barrier to studying TIL subsets [21]. For instance, when tumor cells are grown in traditional 2D culture, they are forced to adapt to an artificially flat, rigid plastic surface. This environment causes them to lose their native three-dimensional morphology, which in turn drives profound changes in gene expression and signaling pathways related to cell adhesion and mechanotransduction [30]. Critically, these changes can lead to an “antigenic shift,” where the expression levels of key tumor-associated antigens and stress ligands recognized by immune cells are significantly altered compared to the original tumor, making 2D models unreliable for assessing T-cell reactivity [31]. These simplified experimental models often lack critical tissue structures and essential immune–tumor interactions, thereby restricting accurate evaluation of γδ T-cell function in situ [21]. Similarly, in vivo animal models, despite their physiological relevance, frequently do not exhibit the full spectrum of human-specific tumor antigens, immune cell subsets, and complex immune responses, resulting in discrepancies between preclinical and clinical outcomes [32–34]. Furthermore, animal studies are often resource intensive, expensive, and constrained by ethical considerations [35]. Collectively, these limitations underscore the importance of developing advanced, human-relevant models to more accurately assess γδ T-cell functionality in clinically relevant contexts.

Patient-derived tumor organoids (PDTOs) have emerged as powerful alternatives for overcoming these limitations because they are high-fidelity models that extensively recapitulate key features of the parental tumors from which they are derived. Comprehensive studies have demonstrated that these 3D cultures maintain the original histopathological architecture, including tissue polarity and cell-to-cell interactions that are lost in 2D culture [36, 37]. Furthermore, they are genomically and transcriptomically stable, preserving the specific mutational landscape, copy number variations, and gene expression signatures of the primary tumor, even across multiple passages [32]. Critically for breast cancer research, they retain the expression of key clinical biomarkers such as the estrogen receptor (ER), progesterone receptor (PR), and HER2, thus reflecting the patient’s specific molecular subtype [28]. This preservation extends to intratumoral heterogeneity, meaning that the diverse subclonal populations present in the original tumor are often maintained within the organoid culture—a feature largely absent from traditional cell lines. The functional consequence of this fidelity is that PDTOs can often predict a patient’s clinical response to therapies, validating them as a robust platform for their effective coculture with immune cells [36]. Immune cell–PDTO coculture systems allow the real-time visualization of immune cell–tumor interactions, quantification of cytotoxic responses, and multiplexed cytokine profiling [37], offering a physiologically relevant context for detailed evaluation of immune cell functionality. For instance, pioneering studies successfully generated tumor-reactive T cells by coculturing organoids with peripheral blood mononuclear cells (PBMCs) [38]. However, this approach relies on activating T cells from the circulation, which may not represent the repertoire or functional state of lymphocytes that have already infiltrated the tumor. This leaves a critical gap in our understanding of the autologous TIL response, particularly for understudied populations like γδ T cells, and simultaneously highlights an urgent clinical need for a robust and reliable platform to evaluate the potential efficacy of a patient’s own TIL therapy before its administration.

Building upon this approach, we established an integrated coculture system of patient-derived breast cancer organoids (BCOs) and autologous or allogeneic immune cells. This system was designed to (i) compare tumor-specific activation between γδ T cells within autologous TIL populations and conventional αβ T cells and (ii) evaluate the potential of healthy donor-derived allogeneic Vγ9Vδ2 T cells to infiltrate, recognize, and eliminate BCOs. Through the incorporation of matched TIL populations, live-cell fluorescence tracing, and multiplex cytokine analyses, our novel coculture system enables the precise functional characterization of γδ T-cell responses within a near-native TME. Collectively, these advancements provide a powerful and translationally relevant model to accelerate the development of γδ T cell-based immunotherapies for solid tumors. Using this platform, we show that autologous γδ T cells exhibit superior tumor reactivity compared to conventional T cells and that allogeneic Vγ9Vδ2 T cells are potently cytotoxic against patient-derived BCOs.

## Materials and methods

### Tissue processing for the establishment of BCOs

Breast cancer and adjacent normal tissue samples were obtained during surgery [39]. Small pieces (approximately 1–4 cm^3^) were promptly cut, placed in tubes on ice and transported to the laboratory within 1 hour after excision. Upon collection, the tissue samples were preserved in Advanced DMEM/F12 (Invitrogen, Waltham, MA, USA) supplemented with GlutaMAX (1×, Gibco, Grand Island, NY, USA), HEPES (1×, Gibco), 3% penicillin/streptomycin, and 10 μM Y-27632 (Rho-associated kinase inhibitor, Abmole, Houston, TX, USA) (collectively referred to as Ad-DF+++ medium).

Upon arrival at the laboratory, the tissues were photographed, weighed, measured in volume, washed three times with cold HBSS containing antibiotics (Gibco) and sectioned into smaller fragments using sterile blades. The minced tissue was then incubated in 10 mL of Ad-DF+++ medium containing 1–2 mg/mL collagenase/DNase I (Sigma-Aldrich, St. Louis, MO, USA) at 37 °C for 1–2 hours with gentle agitation on an orbital shaker. Following enzymatic digestion, the tissue suspension was further dissociated by sequential pipetting and filtered through a 100 μm strainer; any retained tissue was homogenized with a syringe and then washed with medium to ensure complete dissociation. The filtrate was then passed through a 37 μm reversible strainer (Stemcell, Vancouver, BC, Canada, cat. 27215), and the outside of the strainer was washed with PBS to collect cell clusters ranging in size from 40 to 100 μm. The cluster-containing solution was centrifuged at 400 ×g for 5 minutes. Erythrocytes, if present, were lysed using 2 mL of red blood cell lysis buffer (Roche, Basel, Switzerland) for 5 minutes at room temperature, followed by another centrifugation step.

### Organoid culture

The cell cluster pellet was resuspended in growth factor-reduced basement membrane extract (Matrigel; Corning, Corning, NY, USA, 354230) on ice [40]. Forty microliters of the cell-matrix suspension was added to the center of each well of a prewarmed 24-well culture plate. The droplets were allowed to solidify at 37 °C for 15–30 minutes. Once gelation was completed, 400–500 µL of specific organoid culture medium (composition detailed in Supplementary Table 1) was added to each well, and the plate was transferred to a humidified incubator at 37 °C in 5% CO_2_. The medium was changed every 3–4 days.

### Organoid passaging

The organoids were harvested when they reached an appropriate size or confluency [41]. The medium was aspirated, and the Matrigel domes were gently disrupted with a pipette to release the organoids. The disrupted Matrigel-containing organoids were transferred to a 15 mL conical tube, and 5 mL of ice-cold PBS was added. The tube was subsequently centrifuged at 300 ×g for 5 minutes at 4 °C. After the supernatant was aspirated, the pellet was resuspended in 1–2 mL of prewarmed TrypLE Express (Gibco) and incubated at 37 °C for 5–10 minutes to dissociate the organoids. The suspension was gently pipetted to further dissociate the organoids into smaller clusters or single cells. The dissociation was stopped by the addition of 10 mL of cold Advanced DMEM/F12, followed by centrifugation at 300 ×g for 5 minutes. The supernatant was aspirated, and one-fourth of the cell cluster pellet was resuspended in fresh Matrigel and replated as described earlier. The organoids were passaged every 1–2 weeks.

#### Organoid cryopreservation

Every passage, three-fourths of each digested cell cluster was cryopreserved. Cryopreservation was performed by resuspending the digested organoids in freezing medium (90% FBS, 10% DMSO) and slowly freezing them at −80°C before transferring them to liquid nitrogen for storage. For reculturing, the organoids were quickly thawed, washed in cold medium to remove DMSO, and reembedded in the appropriate matrix before being further cultured.

#### Histological staining

The organoids were gently dissociated from the Matrigel to preserve their structure and fixed in 4% paraformaldehyde for 24 hours at room temperature. After fixation, the organoids were washed in PBS, resuspended in 1–2% melted agarose and solidified on ice to maintain their 3D architecture during subsequent processing. The agarose-embedded organoids were then dehydrated in graded ethanol solutions, embedded in paraffin, and sectioned (3–4 µm) for staining and immunohistochemistry (IHC). For histological evaluation, the sections were deparaffinized in xylene, rehydrated in graded alcohol solutions, and stained with hematoxylin and eosin (H&E). The slides were dehydrated, cleared, and mounted for morphological assessment under a microscope [38].

### Immunohistochemistry

For IHC, antigen retrieval was performed in EDTA solution (pH 8.0) under controlled heating. Endogenous peroxidase activity was blocked using 3% hydrogen peroxide, followed by incubation with Novocastra Protein Block (0.4% casein in PBS). The sections were incubated overnight at 4 °C with primary antibodies against estrogen receptor (ER; Abcam, Cambridge, UK, ab16662, 1:200), progesterone receptor (PR; Thermo Fisher Scientific, Waltham, MA, USA, 604–970, 1:200), and human epidermal growth factor receptor 2 (HER2, Thermo Fisher Scientific, MA5– 16,348, 1:100). Detection was performed with fluorophore-conjugated secondary antibodies for immunofluorescence or the Ventana UltraView Universal DAB Detection Kit or EnVision + Rabbit HRP System for chromogenic IHC, and the nuclei were counterstained with hematoxylin. Imaging was carried out using a Zeiss LSM 880 confocal microscope (Carl Zeiss AG, Oberkochen, Germany) or a Zeiss Axioscope A1 microscope equipped with an Axiocam 503 color camera (brightfield). Whole-slide scanning was performed using a D-sight brightfield scanner (Menarini Diagnostics, Florence, Italy) for detailed pathological analysis.

### TIL expansion

TILs were expanded from tumor fragments dissected into approximately 1–8 mm^3^ pieces. The fragments were briefly digested with trypsin (5–10 minutes at 37 °C, 5% CO_2_), washed with PBS, and placed into the individual wells of a 24-well plate (Costar, Corning, NY, USA) containing 2 mL of complete medium (CM) supplemented with 6000 IU/mL IL-2 (Chiron Corp., Emeryville, CA, USA) [42]. The cultures were maintained under standard conditions to support TIL proliferation in vitro.

### Autologous organoid–lymphocyte coculture

To study the interactions between TILs and tumor organoids, we established a coculture system. TILs were thawed and cultured overnight in the presence of 150 U/mL IL-2 to promote activation. Concurrently, tumor organoids were stimulated with 200 ng/mL IFNγ to enhance antigen presentation. The following day, the organoids were dissociated into single cells, and activated TILs were seeded at an effector-to-target (E:T) ratio of 20:1, a high ratio selected to maximize the chances of detecting tumor-reactive T-cell clones, which can be rare within the bulk TIL population. The coculture was performed in the presence of anti-IL-2 and anti-PD-1 antibodies; the anti-IL-2 antibody prevents the preferential growth of non-specific T cells, while the anti-PD-1 antibody blocks inhibitory signals to enhance the activation of tumor-specific TILs.

### Flow cytometry

For our analysis of T-cell reactivity within the coculture system, we began by gating on single cells on the basis of forward scatter (FSC) and side scatter (SSC) characteristics to exclude debris and nonviable cells (Supplementary Figure 1). Then, TILs were identified on the basis of their specific light scatter properties and the exclusion of doublet populations. We then identified CD4^+^ and CD8^+^ T cells within the TIL population using specific surface markers. CD4^+^ T cells were identified using an anti-CD4 antibody (BD, Franklin Lakes, NJ, USA, PE-Cy^TM^7 Mouse Anti-Human CD4, 560649), while CD8^+^ T cells were identified with an anti-CD8 antibody (BD, APC Mouse Anti-Human CD8, 570892). γδ T cells were distinguished by the presence of the γδ T-cell receptor (TCR); specifically the Vγ9Vδ2 subset was detected using a PE-conjugated anti-human Vδ2 TCR antibody (BD, PE Mouse Anti-Human Vδ2 TCR, 555739). Finally, we assessed the percentage of cells positive for CD137, a marker associated with T-cell activation, among the CD4^+^, CD8^+^, and γδ T-cell populations. CD137 was detected using a PE-Cy^TM^5-conjugated anti-human CD137 antibody (BD, PE-Cy^TM^5 Mouse Anti-Human CD137, 551137). The percentage of CD137-positive cells was determined for each subset under both coculture and monoculture conditions to evaluate the impact of the TME on T-cell activation. To quantify the relative increase in the percentage of CD137^+^ cells upon coculture (Fig. 3C), the following formula was used for each T-cell subset (CD8^+^, CD4^+^, and γδ T cells): increase in % of CD137^+^ cells=% of CD137^+^ cells under coculture conditions−% of CD137^+^ cells under TIL-alone conditions.

### Allogeneic γδ T cell expansion

Human peripheral blood mononuclear cells (PBMCs) were isolated from the fresh buffy coats of healthy donors using density gradient centrifugation with Ficoll-Paque (GE Healthcare, Chicago, IL, USA). The cells were then cultured at a concentration of 1 × 10^6^ viable cells/mL in RPMI 1640 medium supplemented with 10% heat-inactivated human AB serum and antibiotics in the presence of zoledronate (50 μM; Sigma) and recombinant human IL-2 (100 IU/mL; Beijing Four Rings Bio-Pharm Co., Beijing, China) [43]. The cultures were maintained at 37°C in 5% CO_2_, and viable cells were counted and replated every other day to maintain the optimal cell concentration (0.5–1.0 × 10^6^ cells/mL). After 12–14 days of culture, the cells were assessed for purity by flow cytometry and subsequently used in experiments.

### Allogeneic organoid–γδ T cell coculture

For the coculture experiments, BCOs and Vγ9Vδ2 T cells were combined at varying E:T ratios to simulate different immunological scenarios. Specifically, we used E:T ratios of 0:0, 1:1, 5:1, and 10:1 to cover a range from no T cells to a substantial excess of T cells. These varying conditions allowed us to assess the dose-dependent effects of Vγ9 Vδ2 T cells on BCOs and to understand the dynamics of T-cell–tumor interactions under various immunological conditions. The cocultures were incubated at 37 °C in 5% CO_2_ for a period of 24 hours to allow sufficient interaction between the BCOs and Vγ9Vδ2 T cells. The coculture medium was replaced every 2–3 days, and the TILs were harvested, counted, and reseeded with fresh organoids weekly to maintain immune activity and evaluate lymphocyte-mediated cytotoxicity within the TME.

### Analysis of the cytotoxicity of γδ T cells upon coculture with tumor organoids

To investigate the cytotoxic potential of γδ T cells against tumor organoids, we first collected γδ T cells with a purity exceeding 85% after ex vivo expansion. These cells were pelleted by centrifugation at 1000 rpm for 5 minutes. The supernatant was discarded, and the γδ T-cell pellet was resuspended in staining solution containing 0.5 μM CellTracker Red CMTPX Dye (Thermo Fisher Scientific) for 45 minutes at 37 °C in 5% CO_2_. After staining, the γδ T cells were washed twice by centrifugation at 300 ×g for 5 minutes in PBS to remove the unbound dye. The cells were then resuspended in culture medium for counting to ensure accurate cell dosing. The γδ T cells were seeded into 96-well plates containing tumor organoids at predetermined E:T ratios of 0:1, 1:1, 5:1, and 10:1. Coculture was initiated in the presence of CellTox Green Cytotoxicity Assay reagent (Promega, Madison, WI, USA, 1:1000 dilution) in a total volume of 200 μl per well. The plates were incubated at 37 °C in 5% CO_2_ for 24 hours. Brightfield and fluorescence images were captured after 0, 6, 12, and 24 hours of coculture to visually track cell interactions. Green fluorescence indicated dead cells, while red fluorescence indicated γδ T cells, allowing the observation of cytotoxic effects and interactions between the organoids and γδ T cells. The green fluorescence intensity was measured every 6 hours to quantify cytotoxic activity.

### Measurement of cytokine levels

To evaluate the activation status of Vγ9 Vδ2 T cells in response to coculture with BCOs, we used a cytometric bead array (CBA) to quantify the levels of key activation markers. After 24 hours of coculture, supernatants were collected for each condition and analyzed with a CBA to measure the concentrations of cytokines and effector molecules with established roles in T-cell-mediated immune responses and relevance to antitumor activity, namely, tumor necrosis factor α (TNF-α, LEGENDplex^TM^ Human TNF-α Capture Bead A10), interferon γ (IFN-γ, LEGENDplex^TM^ Human IFN-γ Capture Bead B4), granzyme B (LEGENDplex^TM^ Human Granzyme B Capture Bead B6), and perforin (LEGENDplex^TM^ Human Perforin Capture Bead B7). The CBA was used according to the manufacturer’s instructions (LEGENDplex^TM^ Human Inflammation Panel, BioLegend, San Diego, CA, USA). In brief, capture beads coated with antibodies specific to each cytokine of interest were mixed with the supernatant samples and incubated to allow for cytokine binding. Following incubation, the detection reagents were added, and the samples were run on a flow cytometer capable of identifying the distinct bead populations on the basis of their fluorescence characteristics. The intensity of fluorescence provided a quantitative measure of the cytokine concentration, which was determined using a standard curve generated from known cytokine concentrations.

### Statistical analysis

All experiments were performed in triplicate, and the results are presented as the mean ± standard deviation. Statistical analysis and data visualization were performed using GraphPad Prism v8.0 software. Student’s t test was used to compare data between two groups, and one-way ANOVA was used to compare data among multiple groups. A *p* value of less than 0.05 was considered to indicate statistical significance.

### Human specimens and bioethical approval

The collection of patient tissues for the generation of tumor organoids and TILs was approved by the Ethical Review Board of The First Affiliated Hospital of Jinan University. All the experimental protocols were conducted in strict accordance with institutional guidelines. The main inclusion criteria were patients with clinically advanced or metastatic or breast cancer, patients aged 18 years or older, patients for whom samples were confirmed to be tumor or normal tissue on the basis of histopathological assessment. Eligible candidates were thoroughly evaluated, and informed consent was obtained prior to surgery.

## Results

### Generation of BCOs

**Fig. 1:**
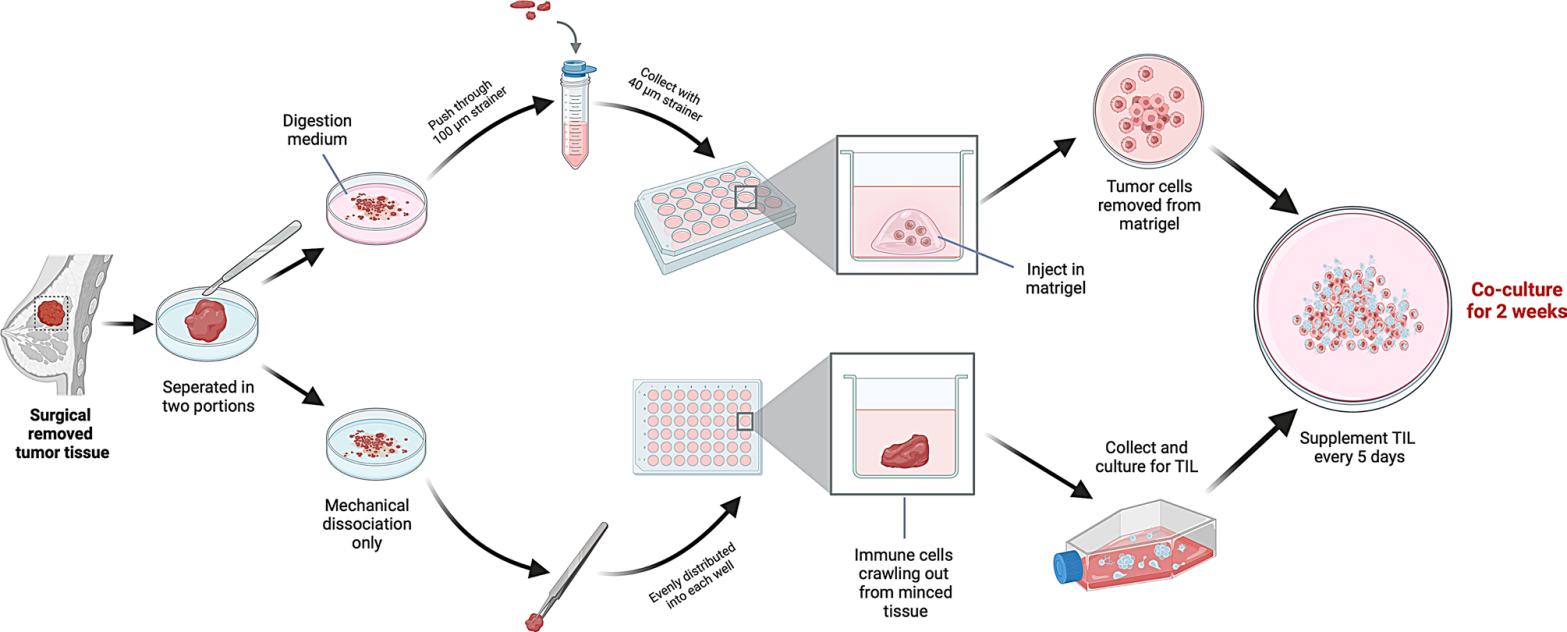
Workflow of autologous tumor organoid–immune cell coculture. This schematic illustrates the stepwise procedure for establishing a tumor organoid–immune coculture system from surgically resected breast cancer tissues. First, tumor tissue was collected and processed into two portions. One portion underwent enzymatic digestion followed by sequential filtration using 100 μm and 40 μm strainers to isolate tumor cell clusters, which were subsequently embedded in Matrigel for 3D organoid culture. The second portion was mechanically dissociated and plated as intact tissue fragments, allowing tumor-infiltrating leukocytes (TILs) to migrate out into the culture medium. These TILs were collected and expanded for further analysis. For coculture experiments, tumor organoids were removed from Matrigel and combined with autologous TILs at defined effector-to-target (E:T) ratios. The coculture was maintained for two weeks, with fresh TILs being added every five days to sustain immune activation. This model enabled the assessment of tumor-reactive immune responses in breast cancer within a physiologically relevant microenvironment

**Fig. 2:**
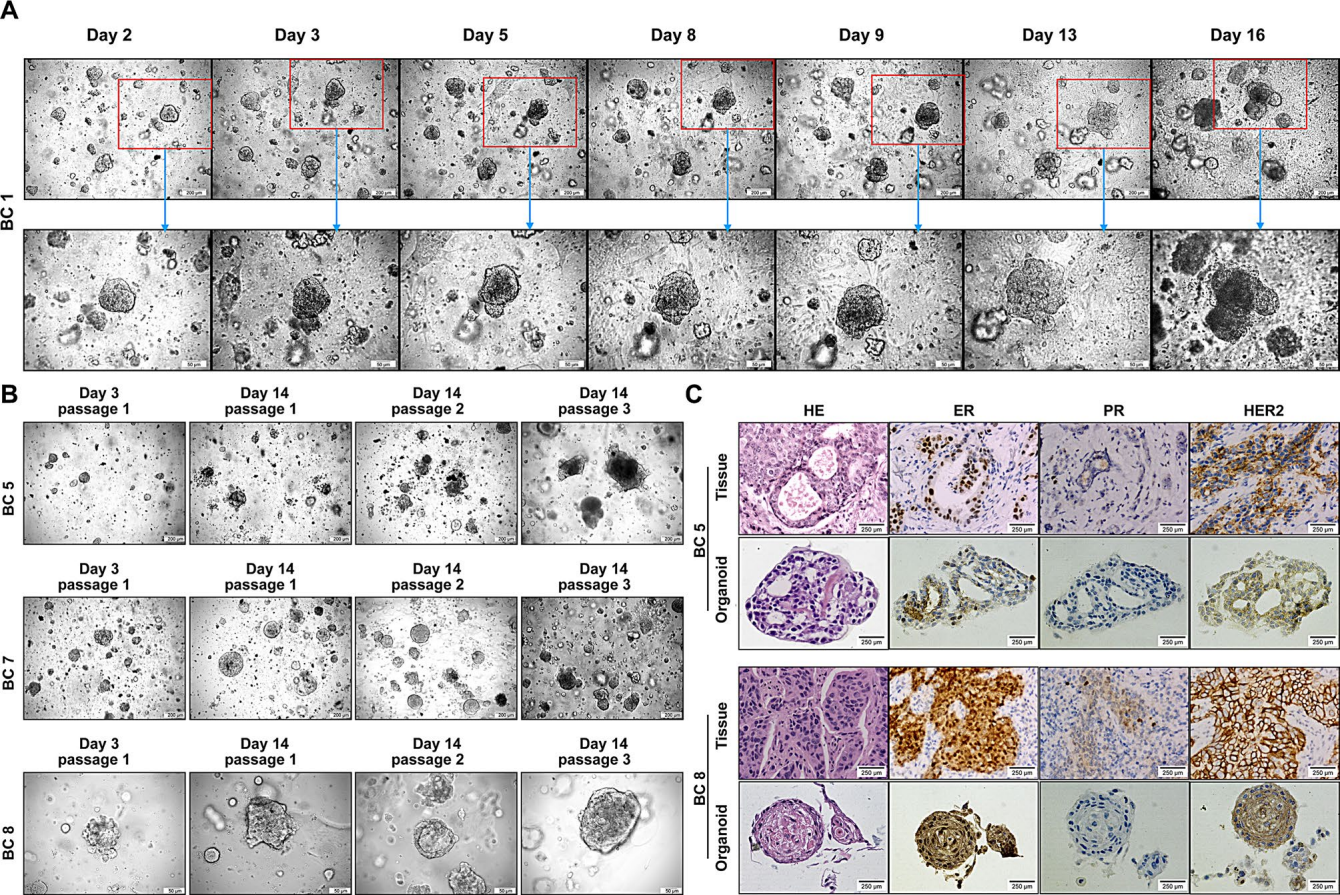
Establishment and characterization of breast cancer organoids (BCOs). (**A**) Time-lapse imaging of BCO growth (BC1) over a 16-day culture period. The organoids increased in size and displayed progressive morphological changes, including the expansion of cell masses. The lower panels are magnified images of regions from the upper panels. Scale bars: 200 μm (top) and 50 μm (bottom). (**B**) Passaging of BCOs. Organoids were enzymatically dissociated and passaged at a 1:2 ratio, maintaining stable growth across three passages. Scale bars: 200 μm (BC5 and BC7) and 50 μm (BC8). (**C**) Histological and immunohistochemical analysis of breast cancer organoids and corresponding primary tumor tissues. Hematoxylin & Eosin (H&E) staining and immunostaining for estrogen receptor (ER), progesterone receptor (PR), and HER2 demonstrating that the organoids retained the expression of key tumor markers. Scale bar: 250 μm

To establish an immune cell–tumor organoid coculture system for studying the function and potential therapeutic efficacy of γδ T cells in the context of cancer, we first generated patient-derived BCOs using a standardized and reproducible approach (Fig. 1). Our novel workflow, which addresses the common challenge of isolating high-quality organoid-forming units, involved the enzymatic digestion of tumor tissues, followed by sequential filtration through 100 μm and 40 μm strainers. The 100 μm filtration step was crucial for removing large undigested tissue fragments that had not been sufficiently broken down during enzymatic digestion. After the filtrate was collected, the tissues were passed through a 40 μm filter to isolate small, cohesive clusters with high organoid-forming potential while filtering out single cells with poor organoid-forming potential. This optimized filtration strategy represents a key advance for consistently enriching the ideal starting material for robust organoid growth. These clusters were then embedded in Matrigel, a basement membrane matrix that mimics the in vivo extracellular environment and supports three-dimensional growth, which is crucial for retaining key tumor features. Through this approach, highly viable and tumor-representative organoids that appeared as a solid structure with multiple cell layers, accurately reflecting the complex architecture and histological characteristics of in vivo tumors, were obtained (Fig. 2).

### Characterization of BCOs

To observe organoid growth without passaging, we imaged the organoids with a microscope over a 16-day culture period. Microscopy revealed that organoids derived from patient tumors (e.g., BC11, Sup F2) exhibited markedly faster growth, approximately doubling in diameter every 4–5 days, while normal organoids showed minimal size increase over the same 16-day period; this rapid expansion led to morphological changes in the BCOs, such as tumor budding (Fig. 2A). Thus, the cancer organoids displayed robust expansion, suggesting their intrinsic proliferative capacity when cultured in a 3D matrix. In parallel, as a control, we established normal breast tissue-derived organoids (Supplementary Figure 2A). Furthermore, the normal organoids exhibited a uniform sphere/lumen-like (acini-like) structure and well-defined tubular (terminal duct-like) formations, whereas tumor-derived organoids displayed a more compact, solid structure with prominent solid internal contents (Supplementary Figure 2A). These data indicate that normal organoids retain healthy breast structure, BCOs have malignant traits—validating our models as suitable for γδ T cell-tumor interaction studies.

During long-term culture, the enlargement of tumor organoids may lead to the rupture of the Matrigel, enable the organoids to adhere to the culture surface (data not shown). Therefore, to maintain organoid growth, the breast organoids were passaged at a 1:2 ratio. Long-term stability was assessed by monitoring consistent morphology and growth across multiple passages. Specifically, the structural integrity and growth of the organoids from patients BC5, BC7 and BC8 were maintained through passages 1 to 4 (Fig. 2B), demonstrating the reliability of the model for long-term studies.

Several studies have shown that BCOs can maintain similar histopathological and molecular features as the original tumor [22, 44, 45]. To confirm that the BCOs faithfully recapitulated the features of the parent tumors, we performed histological and immunohistochemical analysis on sections of the organoids and the original tumor tissue. H&E staining revealed that the organoids maintained a solid, multi-layered architecture consistent with the malignant morphology seen in the primary tumor sections (Fig. 2C). Critically, IHC analysis demonstrated that the expression profiles of the clinical biomarkers ER, PR, and HER2 were consistently maintained in the BCOs. This was observed across multiple patient-derived lines; for example, organoids from patients BC5 and BC8 accurately mirrored the biomarker expression levels of their corresponding primary tumors (Fig. 2C, right panel). Furthermore, organoids derived from patient BC5 exhibited high ER and PR expression and moderate HER2 expression, consistent with that patient’s tumor profile. This validated consistency in histopathology and patient-specific molecular features across different samples underscores the reliability of our BCOs as a clinically relevant model for subsequent immunological studies.

### γδ T cells constitute a tumor-reactive subset within TILs

Tumor-infiltrating γδ T cells have been linked to favorable outcomes across multiple solid malignancies, including breast cancer; however, the fraction of γδ T cells that is functionally tumor reactive within the heterogeneous TIL pool remains poorly defined in breast cancer [27, 46]. To address this, we expanded autologous TILs in parallel with patient-derived BCOs by culturing tumor tissue fragments in high-dose IL-2 for 19 days. During culture, lymphocytes progressively emigrated from tumor fragments, transitioning from early outgrowth (Day 5) to the formation of proliferative clusters (Day 15) and larger aggregates (Day 19) (Supplementary Figure 2B), yielding a viable TIL product for downstream functional characterization.

**Fig. 3:**
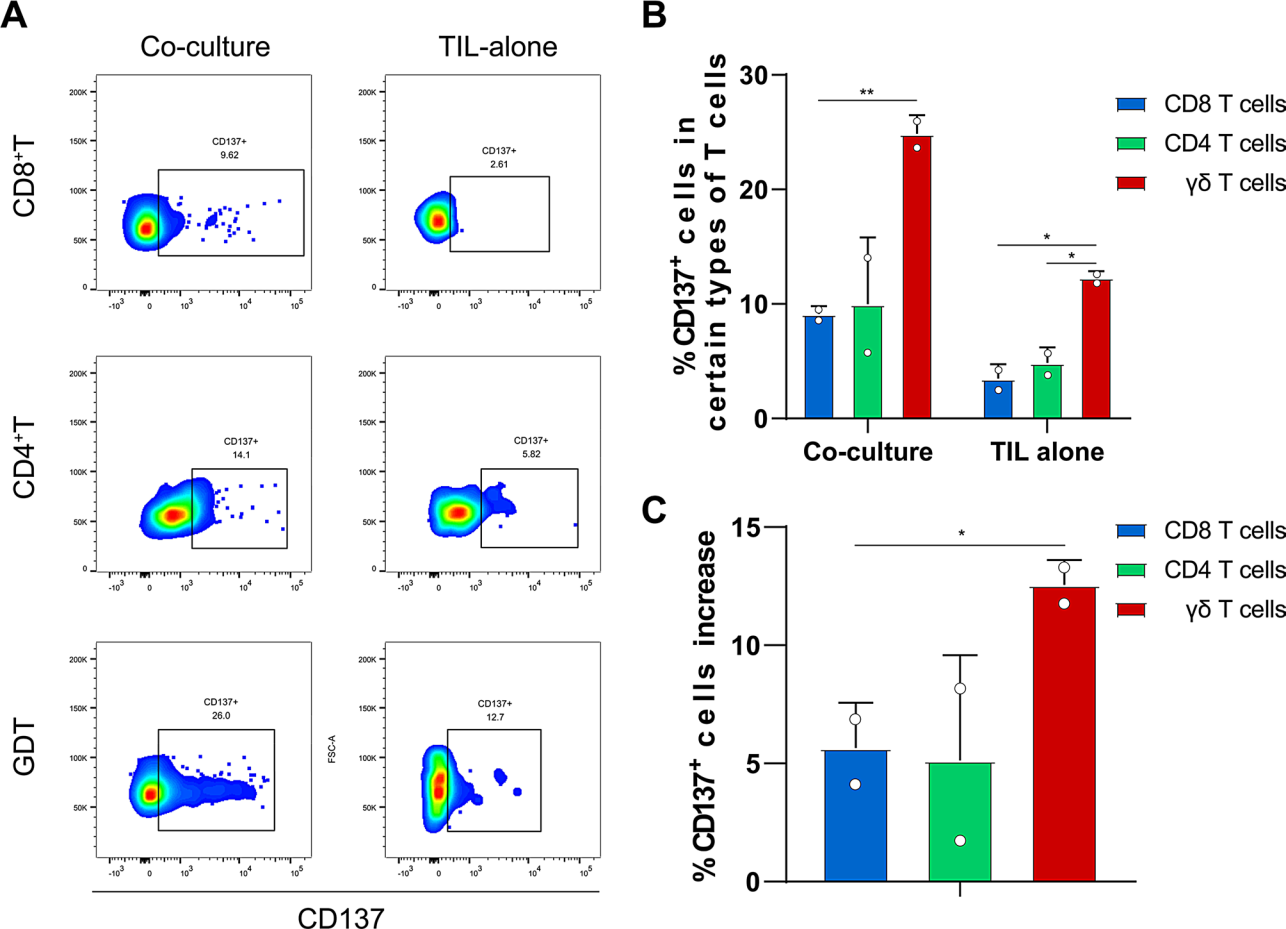
Induction of tumor reactivity among different types of tumor-infiltrating leukocytes (TILs) by coculture with autologous tumor organoids. (**A**) Flow cytometry analysis of CD137 expression in different subsets of TILs after coculture with autologous tumor organoids. Representative flow cytometry plots display the percentage of CD137-expressing (activated) cells among CD8^+^ T cells, CD4^+^ T cells, and γδ T cells under two conditions: coculture with autologous tumor organoids (left panels) and culture of TILs alone (right panels). The proportion of CD137^+^ cells notably increased under coculture conditions, particularly for the γδ T-cell subset. (**B**) Quantification of the percentage of CD137^+^ TILs within different cell subsets. Bar graph showing the percentage of CD137^+^ cells within each T-cell subset (CD8^+^, CD4^+^, and γδ T cells) after coculture with tumor organoids (left) or culture of TILs alone (right). γδ T cells exhibited the most significant induction of CD137 expression under coculture conditions, followed by CD4^+^ and CD8^+^ T cells. The data are presented as the mean ± standard deviation (SD). (**C**) Relative increase in the percentage of CD137^+^ T cells after coculture. Bar graph depicting the net increase in the percentage of CD137^+^ cells within each TIL subset following coculture with tumor organoids compared with culture of TILs alone. The greatest induction of CD137 expression was observed in the γδ T-cell population, suggesting increased tumor reactivity in response to the autologous tumor organoid microenvironment

To functionally assess tumor reactivity within the TIL compartment, we used CD137 (4-1BB) as the primary activation marker. CD137 is rapidly induced on T cells following antigen-specific stimulation and has been widely used to detect and enrich tumor-reactive T cells in tumor organoid co-culture systems and T cell receptor discovery studies [47, 48]. In the TIL-alone group, flow cytometric analysis revealed that 12.3% of γδ T cells were CD137^+^, consistent with prior reports that only a subset of bulk TILs is tumor reactive while a substantial fraction can be non–tumor-reactive/bystander cells [49]. Notably, the frequency of CD137^+^ cells within the γδ T-cell subset was higher than in CD8^+^ T cells (3.49%, *p* = 0.0118) and CD4^+^ T cells (4.86%, *p* = 0.0190) in TIL fragments (Fig. 3A,B). Collectively, these data indicate that γδ TILs contain a CD137^+^ tumor-reactive subset and support their contribution to anti-tumor immunity alongside αβ T cells.

### Autologous breast cancer organoid–TIL coculture preferentially enhances tumor-reactive γδ T cells

To test whether autologous tumor organoids can enhance tumor-reactive activation within TIL subsets, we cocultured matched breast cancer organoids (BCOs) with autologous TILs at a defined effector-to-target (E:T) ratio and maintained the cultures for two weeks with weekly stimulation using autologous tumor organoids (Fig. 1).

Following coculture with autologous BCOs, representative flow plots showed increased CD137^+^ populations across multiple T-cell subsets (Fig. 3A). Quantification confirmed CD137 upregulation in cocultured TILs compared with the TIL-alone condition (Fig. 3B). Notably, γδ T cells reached 24.85% CD137^+^ under coculture conditions, and the CD137^+^ fraction within γδ T cells was significantly higher than that in CD8^+^ T cells (24.85% vs. 9.15%, *p* = 0.0062) and numerically higher than that in CD4^+^ T cells (24.85% vs. 9.99%, *p* = 0.0736) (Fig. 3B). When we subtracted the baseline values of the TIL-alone group from those of the coculture group to calculate the fold increase in CD137^+^ positivity, we found that the increase in γδ T cells was significantly greater than that in CD8^+^ T cells (12.55% vs. 5.66%, *p* = 0.0467), whereas the difference relative to CD4^+^ T cells did not reach statistical significance (12.55% vs. 5.125%, *p* = 0.1492) (Fig. 3C). Collectively, these data indicate that autologous BCO–TIL coculture enhances tumor-reactive activation and does so most prominently within the γδ T-cell compartment.

### A fluorescent coculture system reveals the cytotoxicity of allogeneic Vγ9 Vδ2 T-cell to BCOs

**Fig. 4:**
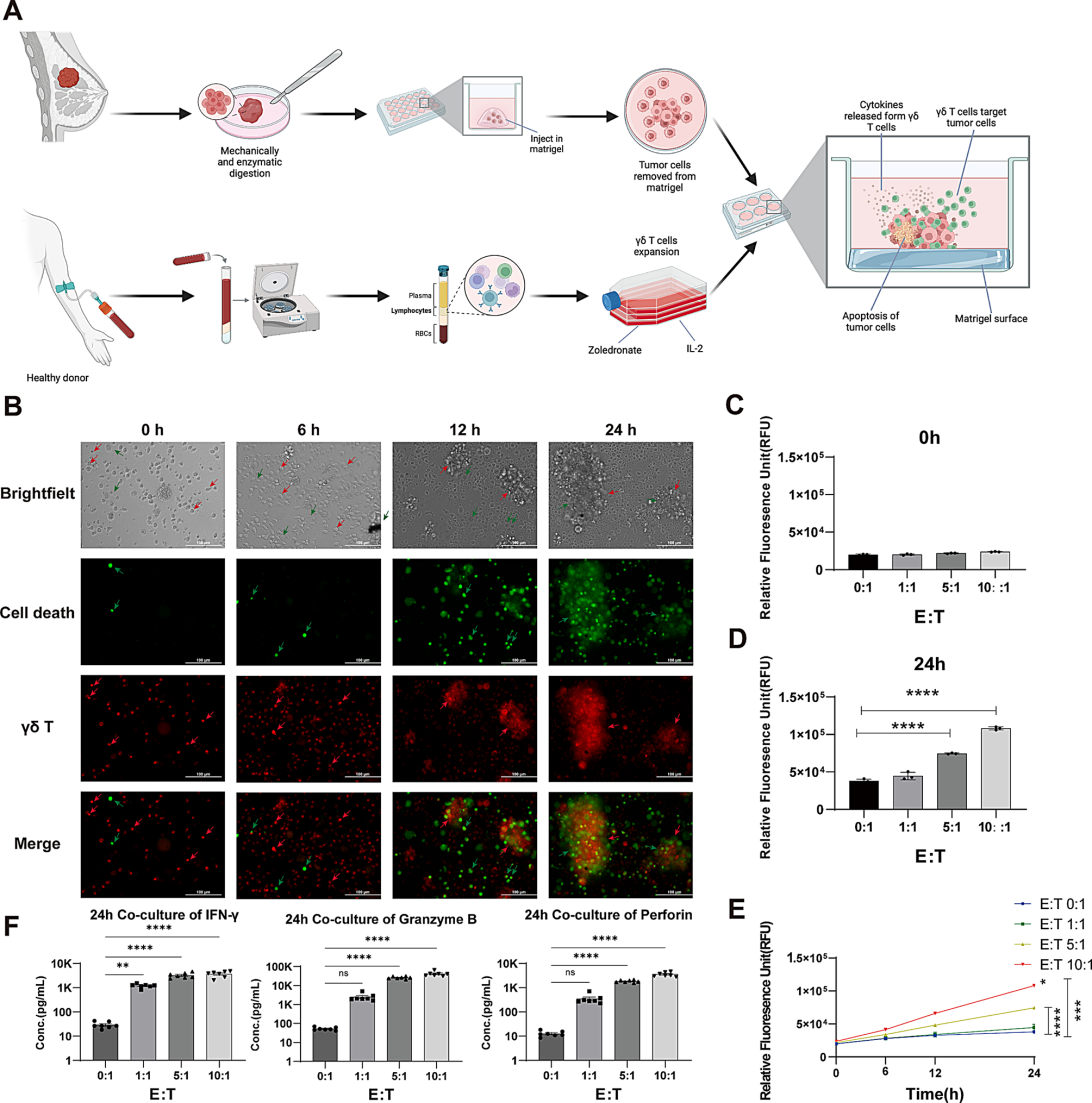
Allogeneic γδ T-cell-mediated cytotoxicity against breast cancer organoids (BCOs). (**A**) Workflow of allogeneic γδ T-cell expansion and coculture with breast cancer organoids. Tumor tissues from breast cancer patients were subjected to Mechanical and enzymatic digestion to establish 3D organoid cultures. Peripheral blood mononuclear cells (PBMCs) from healthy donors were isolated and expanded using zoledronate and IL-2, selectively enriching γδ T cells. These γδ T cells were cocultured with breast cancer organoids to assess their cytotoxic effects, including by analyzing cytokine secretion and tumor cell apoptosis. (**B**) Time-lapse imaging of γδ T-cell-mediated cytotoxicity against tumor organoids. Representative images at 0 h, 6 h, 12 h, and 24 h showing tumor organoid morphology (brightfield), apoptotic cell death (green fluorescence, CellTox green), and γδ T-cell infiltration (red fluorescence, CellTracker red). The merged images illustrate the progressive interaction between γδ T cells and tumor cells, leading to increased cell death over time. Scale bar: 100 μm. E:T = 10:1. (**C–D**) Quantification of tumor cell death at different effector-to-target (E:T) ratios. Relative fluorescence unit (RFU) values at 0 h (**C**) and 24 h (**D**) showing a significant increase in cell death at higher E:T ratios (0:1, 1:1, 5:1, and 10:1), confirming γδ T-cell-mediated cytotoxicity. Statistical significance: ****p < 0.0001. (**E**) Kinetics of tumor cell death over time. RFU values measured at 0 h, 6 h, 12 h, and 24 h demonstrating a time-dependent increase in tumor cell death, particularly at high E:T ratios. Statistical significance: *p < 0.05; ***p < 0.001; ****p < 0.0001. (**F**) Cytokine secretion in γδ T-cell–tumor organoid cocultures. Quantification of IFN-γ, granzyme B, and perforin levels at 24 h for different E:T ratios by a cytometric bead array (CBA). γδ T cells released large amounts of IFN-γ, granzyme B, and perforin at elevated E:T ratios, while TNF-α levels remained relatively stable. Statistical significance: ns (not significant), **p < 0.01, ****p < 0.0001

Considering our findings from the autologous TIL coculture system, and given that recent research has demonstrated that the cancer antigens recognized by γδ T cells are mainly phosphoantigens, which bind to BTN3A1 and BTN2A1 within the cytoplasm and induce the exposure of BTN3A1 and BTN2A1 epitopes outside the cell to effectively bind to the Vγ9 Vδ2 TCR and ultimately activate γδ T cells [21, 50], To evaluate this, PBMCs from healthy donors were isolated and stimulated ex vivo with zoledronate and IL-2, leading to the selective expansion of the Vγ9 Vδ2 T-cell subset (Fig. 4A). We then cocultured these enriched Vγ9 Vδ2 T cells with BCOs at various effector-to-target (E:T) ratios (1:1, 5:1, and 10:1) to evaluate their cytotoxic potential. Real-time fluorescence imaging revealed the progressive infiltration of Vγ9 Vδ2 T cells (red fluorescence) into organoid structures and an associated increase in the number of apoptotic cells (green fluorescence) over 24 hours (Fig. 4B). By 24 hours, a significantly higher green fluorescence intensity was observed at the 10:1 E:T ratio than at lower ratios (Figs. 4C–E). These observations confirm that an adequate density of effector cells is crucial for maximizing γδ T cell-mediated cytotoxic responses. At lower E:T ratios (1:1 and 5:1), the cytotoxic effect was less pronounced, confirming that a higher density of effector cells leads to enhanced tumor killing. These results suggest that γδ T cells exert their cytotoxic effect on BCOs in a time-dependent manner, with optimal efficacy achieved after 24 hours of coculture at the highest E:T ratio.

To investigate the mechanism of this cytotoxic response, we used a CBA to measure cytokine and cytotoxic molecule release. Elevated levels of IFN-γ, granzyme B, and perforin were detected in the cocultures at a 10:1 E:T ratio (all *p* < 0.0001; Fig. 4F), highlighting the immune activation and cytotoxic response of γδ T cells.

Taken together, these findings demonstrate that allogeneic Vγ9 Vδ2 T cells can effectively kill patient-derived BCOs in a dose- and time-dependent manner, a response that is mediated by the release of key cytotoxic effector molecules.

## Discussion

Here, we established a high-fidelity autologous platform to rigorously evaluate patient-derived TILs, overcoming the physiological limitations of conventional PBMC-based models. By reconstructing the native immune microenvironment, we reveal that active stimulation by autologous tumor organoids preferentially enhances γδ T cell activation over their αβ counterparts (Fig. 3A–B). Unlike conventional CD8+ T cells, γδ T cells exhibited a superior intrinsic sensitivity to autologous tumor signals, challenging the CD8-centric paradigm and identifying these cells as a potent effector population for future adoptive cell therapies (Fig. 3B). Complementing this biological discovery, we engineered a multi-modal evaluation platform integrating live fluorescent tracing with multiplex cytokine analysis to enable high-resolution, real-time assessment of T-cell infiltration and tumor engagement within a 3D microenvironment (Fig. 4B). Leveraging these analytical capabilities, we substantiated the efficacy of off-the-shelf allogeneic Vγ9 Vδ2 T cells in dismantling patient-derived tumor architecture.

To capture the pre-primed immune landscape, we utilized autologous TILs—a methodological approach that differs from the reliance on peripheral blood lymphocytes (PBMCs) described in foundational organoid models [47, 51]. While PBMC-based systems assess the potential of circulating T-cell repertoires to generate tumor-reactive clones, TIL-based platforms characterize lymphocytes that have already matured within the tumor microenvironment [47, 48]. By expanding TILs directly from tumor fragments, we were able to evaluate populations that possess tissue-resident phenotypes and have undergone antigen-driven selection in situ [46, 49]. This methodology is consistent with the recent application of TIL–organoid co-cultures for tumor-specific TCR identification [48]. Furthermore, the inclusion of a TIL-alone control group served as an internal benchmark for specificity, distinguishing tumor-driven activation from the non-specific bystander responses identified in human solid tumors [52]. This experimental design ensures that the cellular reactivity observed in our 3D microenvironment is a functional consequence of tumor engagement rather than an artifact of the expansion process.

Our characterization of the ex vivo TIL compartment revealed that γδ T cells represent a distinct, naturally primed effector subset within the breast cancer immune landscape (Fig. 3). The observation that γδ TILs exhibit a notably higher baseline frequency of activation-marker expression compared to conventional CD8^+^ or CD4^+^ subsets provides functional support for the molecular insights of Janssen et al. [53](Fig. 3). While their work utilized archival material to establish that breast cancer-derived γδ TCRs possess intrinsic antitumor potential at the genetic level, our study provides live-cell functional evidence that these cells exist in a state of heightened reactivity even before formal tumor challenge (Fig. 3). This baseline activation aligns with the concept of “antigen-experienced” TILs and underscores the challenge posed by the “bystander” effect identified by Duhen et al., where a substantial fraction of the T-cell infiltrate lacks tumor-specific functionality [52]. By identifying γδ T cells as the most reactive subset in the expanded TIL product, we highlight an underappreciated effector reservoir that appears to be naturally sensitized by the native breast tumor microenvironment (Fig. 3).

Beyond their baseline reactivity, the disproportionate enhancement of γδ T cells within the autologous BCO coculture highlights a superior intrinsic sensitivity to autologous tumor signals (Fig. 3). The magnitude of γδ T-cell activation exceeded that observed in the HLA-restricted αβ compartment, supporting γδ T cells as prominent responders in this patient-matched 3D setting. Notably, this functional potentiation was observed without exogenous agonists, consistent with the possibility that patient-derived organoids preserve endogenous stress-associated cues and/or metabolic signals sufficient to trigger γδ T-cell responses. While foundational organoid-based enrichment studies have largely emphasized the expansion of MHC-restricted CD8+ clones [48, 51], our findings extend this paradigm by demonstrating that, in our patient-matched coculture system, repeated stimulation with autologous tumor organoids disproportionately amplified the tumor-reactive γδ fraction relative to αβ subsets, indicating that γδ T cells constitute a readily inducible effector axis within TIL products (Fig. 3). This expands organoid-based enrichment paradigms beyond a strictly CD8-focused framework and supports prioritizing γδ T cells for downstream adoptive cell therapy development. In line with this, recent clinical evidence has linked tumor-reactive γδ T-cell expansion to complete responses following immune checkpoint blockade [54].

While the preferential activation of autologous γδ TILs underscores their therapeutic potential, translating these findings into scalable, off-the-shelf interventions requires validating universal effector subsets within a robust analytical framework. We prioritized the Vγ9 Vδ2 T cell subset for allogeneic validation due to its MHC-independent recognition of metabolic phosphoantigens through butyrophilin-mediated mechanisms (e.g., BTN3A1/BTN2A1) [54, 55]. This occurs via a proposed “inside-out” signaling process in which dysregulated tumor metabolism leads to intracellular phosphoantigen accumulation, binding to BTN3A1 within tumor cells and inducing conformational changes that are sensed by the Vγ9 Vδ2 T-cell receptor [47, 56]. This recognition profile offers a clinical advantage: because Vγ9 Vδ2 TCRs do not recognize allo-HLA molecules, the risk of graft-versus-host disease (GVHD) is expected to be reduced, supporting development of allogeneic products [55]. To characterize this response, we leveraged our multimodal platform—combining real-time fluorescent-cell tracing with longitudinal multiplex cytokine analysis—to provide a synchronized assessment of spatial infiltration, physical tumor engagement, and effector output (Fig. 4). Our observations of tumor injury together with IFN-γ, granzyme B, and perforin secretion are consistent with prior clinical evidence supporting the safety and biological activity of allogeneic Vγ9 Vδ2 T-cell therapy in late-stage solid tumors [26] (Fig. 4). Ultimately, our system provides a standardized, TME-mimetic framework for rapid preclinical benchmarking of diverse cellular products beyond γδ T cells, including CAR-T, TCR-T, and NK-cell therapies, prior to clinical translation.

While our findings provide functional evidence supporting the antitumor potential of γδ T cells in a 3D organoid coculture system, several limitations remain and merit further investigation. First, our current model is a simplified representation of the TME, as it lacks other critical cellular components such as cancer-associated fibroblasts, endothelial cells, and diverse immunosuppressive populations (e.g., M2 macrophages, Tregs). Furthermore, to bridge the translational gap between in vitro efficacy and in vivo outcomes, a key future direction will be the use of patient-derived organoid xenograft (PDOX) models. These models, which involve implanting organoids into immunodeficient mice co-engrafted with human immune systems, are essential for evaluating trafficking, persistence, and therapeutic efficacy of γδ T cells in a systemic context [41, 57]. As γδ T cells bridge innate and adaptive immunity, they encompass multiple phenotypically and functionally distinct subsets, such as tissue-resident Vδ1 and circulating Vδ3 cells, that may respond differently during tumor progression. In this study, we evaluated γδ T-cell activation at the population level and did not distinguish subset-specific dynamics. To elucidate subset-specific functions, future studies should incorporate high-resolution single-cell transcriptomics and/or spatial multiomic profiling after coculture. These approaches may also reveal interactions between γδ T cells and other lymphocyte populations within the TME. In addition, further engineering of γδ T cells, such as development of CAR-γδ or TCR-γδ constructs, may improve tumor targeting, persistence, and effector function in solid tumors. Finally, our organoid–immune cell coculture system, enabling fluorescent tracing and multiplex cytokine analysis, may serve as a broadly applicable preclinical tool to assess and refine diverse cell-based therapies prior to clinical translation.

## Conclusion

In summary, we established a patient-derived BCO–immune cell coculture system that faithfully recapitulates the histological architecture and biomarker expression of parental tumors. Upon coculture with autologous tumor organoids, we provided direct evidence that autologous γδ T cells exhibit superior tumor-specific activation compared to conventional αβ T cell subsets. Furthermore, we demonstrated that allogeneic Vγ9 Vδ2 T cells mediate potent, dose-dependent cytotoxicity against BCOs, driven by the secretion of IFN-γ and granzyme B. Collectively, this study validates the utility of organoid-based coculture models for dissecting immune-tumor interactions in a near-native TME and provides functional data supporting the antitumor efficacy of γδ T cells.

## Electronic supplementary material

Below is the link to the electronic supplementary material.

Supplementary Material 1

## Data Availability

The raw data generated during this study are available from the corresponding author (Liangping Li) upon reasonable request and through collaborative investigations. All other data needed to evaluate the conclusions in the paper are presented in the paper or the supplementary materials.

## Abbreviations

ACT: Adoptive cell therapy
BCO: Breast cancer organoid
CBA: Cytometric bead array
E: T Effector-to-target ratio
ER: Estrogen receptor
FSC: Forward scatter
HER2: Human epidermal growth factor receptor 2
HLA: Human leukocyte antigen
ICIs: Immune checkpoint inhibitors
IFN-γ: Interferon gamma
IHC: Immunohistochemistry
MHC: Major histocompatibility complex
PBMCs: Peripheral blood mononuclear cells
PDTO: Patient-derived tumor organoid
PR: Progesterone receptor
RFU: Relative fluorescence unit
SSC: Side scatter
TCR: T-cell receptor
TILs: Tumor-infiltrating lymphocytes
TME: Tumor microenvironment
TNF-α: Tumor necrosis factor-alpha

## References

[1] Ali I, Alsehli M, Scotti L, Tullius Scotti M, Tsai S, Yu R, et al. Progress in polymeric nano-medicines for theranostic cancer treatment. Polymers. 2020;12. 10.3390/polym12030598 PMC7182942 32155695

[2] Ali I, Wani WA, Saleem K, Wesselinova D. Syntheses, DNA binding and anticancer profiles of L-glutamic acid ligand and its copper(II) and ruthenium(III) complexes. Med Chem. 2013;9:11–21. 22741786 10.2174/157340613804488297

[3] Ashraf IARK, Rather. Social aspects of cancer genesis. Cancer Ther. 2011;8:6–14.

[4] Imran Ali WAWK. Thalidomide: a banned drug resurged into future anticancer drug. Curr Drug Ther. 2012;13–23.

[5] Imran Ali MNLM. Difnjtusz personal not use distribution only. Curr Med Chem. 2016;2159–87.

[6] Ali I, Wani WA, Khan A, Haque A, Ahmad A, Saleem K, et al. Synthesis and synergistic antifungal activities of a pyrazoline based ligand and its copper(II) and nickel(II) complexes with conventional antifungals. Microb Pathog. 2012;53:66–73. 22575887 10.1016/j.micpath.2012.04.005

[7] Zhang M, Liu C, Tu J, Tang M, Ashrafizadeh M, Nabavi N, et al. Advances in cancer immunotherapy: historical perspectives, current developments, and future directions. Mol Cancer. 2025;24:136. 40336045 10.1186/s12943-025-02305-x PMC12057291

[8] Mitra A, Kumar A, Amdare NP, Pathak R. Current landscape of cancer immunotherapy: harnessing the immune arsenal to overcome immune evasion. Biology (Basel). 2024;13. 10.3390/biology13050307 PMC11118874 38785789

[9] Taylor BC, Balko JM. Mechanisms of MHC-I downregulation and role in immunotherapy response. Front Immunol. 2022;13:844866. 35296095 10.3389/fimmu.2022.844866 PMC8920040

[10] Reck M, Rodriguez-Abreu D, Robinson AG, Hui R, Csoszi T, Fulop A, et al. Pembrolizumab versus chemotherapy for PD-L1-positive non-small-cell lung cancer. N Engl J Med. 2016;375:1823–33. 27718847 10.1056/NEJMoa1606774

[11] Brahmer J, Reckamp KL, Baas P, Crino L, Eberhardt WE, Poddubskaya E, et al. Nivolumab versus docetaxel in advanced squamous-cell non-small-cell lung cancer. N Engl J Med. 2015;373:123–35. 26028407 10.1056/NEJMoa1504627 PMC4681400

[12] Loi S, Michiels S, Adams S, Loibl S, Budczies J, Denkert C, et al. The journey of tumor-infiltrating lymphocytes as a biomarker in breast cancer: clinical utility in an era of checkpoint inhibition. Ann Oncol. 2021;32:1236–44. 34311075 10.1016/j.annonc.2021.07.007

[13] Dagar G, Gupta A, Masoodi T, Nisar S, Merhi M, Hashem S, et al. Harnessing the potential of CAR-T cell therapy: progress, challenges, and future directions in hematological and solid tumor treatments. J Transl Med. 2023;21:449. 37420216 10.1186/s12967-023-04292-3 PMC10327392

[14] Larkin J, Del VM, Mandala M, Gogas H, Arance FA, Dalle S, et al. Adjuvant nivolumab versus ipilimumab in resected stage III/IV melanoma: 5-year efficacy and biomarker results from CheckMate 238. Clin Cancer Res. 2023;29:3352–61. 37058595 10.1158/1078-0432.CCR-22-3145 PMC10472092

[15] Zhao Y, Deng J, Rao S, Guo S, Shen J, Du F, et al. Tumor infiltrating lymphocyte (TIL) therapy for solid tumor treatment: progressions and challenges. Cancers. 2022;14. 10.3390/cancers14174160 PMC9455018 36077696

[16] Kazemi MH, Sadri M, Najafi A, Rahimi A, Baghernejadan Z, Khorramdelazad H, et al. Tumor-infiltrating lymphocytes for treatment of solid tumors: it takes two to tango? Front Immunol. 2022;13:1018962. 36389779 10.3389/fimmu.2022.1018962 PMC9651159

[17] Pu Y, Zhou G, Zhao K, Chen Y, Shen S. Immunotherapy for recurrent glioma-from bench to bedside. Cancers. 2023;15. 10.3390/cancers15133421 PMC10340154 37444531

[18] Eil R, Vodnala SK, Clever D, Klebanoff CA, Sukumar M, Pan JH, et al. Ionic immune suppression within the tumour microenvironment limits T cell effector function. Nature. 2016;537:539–43. 27626381 10.1038/nature19364 PMC5204372

[19] Yu Y, Ho P. Sculpting tumor microenvironment with immune system: from immunometabolism to immunoediting. Clin Exp Immunol. 2019;197:153–60. 30873592 10.1111/cei.13293 PMC6642881

[20] Fisher J, Sharma R, Don DW, Barisa M, Hurtado MO, Abramowski P, et al. Engineering gammadeltaT cells limits tonic signaling associated with chimeric antigen receptors. Sci Signal. 2019;12. 10.1126/scisignal.aax1872 PMC7055420 31506382

[21] Hu Y, Hu Q, Li Y, Lu L, Xiang Z, Yin Z, et al. Gammadelta T cells: origin and fate, subsets, diseases and immunotherapy. Signal Transduct Target Ther. 2023;8:434. 37989744 10.1038/s41392-023-01653-8 PMC10663641

[22] Hayday AC. Gammadelta T cells and the lymphoid stress-surveillance response. Immunity. 2009;31:184–96. 19699170 10.1016/j.immuni.2009.08.006

[23] Bonneville M, O’Brien RL, Born WK. Gammadelta T cell effector functions: a blend of innate programming and acquired plasticity. Nat Rev Immunol. 2010;10:467–78. 20539306 10.1038/nri2781

[24] Chien Y, Meyer C, Bonneville M. Gammadelta T cells: first line of defense and beyond. Annu Rev Immunol. 2014;32:121–55. 24387714 10.1146/annurev-immunol-032713-120216

[25] Li W, Zhao X, Ren C, Gao S, Han Q, Lu M, et al. The therapeutic role of gammadeltaT cells in TNBC. Front Immunol. 2024;15:1420107. 38933280 10.3389/fimmu.2024.1420107 PMC11199784

[26] Xu Y, Xiang Z, Alnaggar M, Kouakanou L, Li J, He J, et al. Allogeneic Vgamma9Vdelta2 T-cell immunotherapy exhibits promising clinical safety and prolongs the survival of patients with late-stage lung or liver cancer. Cell Mol Immunol. 2021;18:427–39. 10.1038/s41423-020-0515-7. 32939032 10.1038/s41423-020-0515-7 PMC8027668

[27] Kabelitz D, Serrano R, Kouakanou L, Peters C, Kalyan S. Cancer immunotherapy with γδ T cells: many paths ahead of us. Cell Mol Immunol. 2020;17:925–39. 32699351 10.1038/s41423-020-0504-x PMC7609273

[28] Sawaisorn P, Gaballa A, Saimuang K, Leepiyasakulchai C, Lertjuthaporn S, Hongeng S, et al. Human Vgamma9Vdelta2 T cell expansion and their cytotoxic responses against cholangiocarcinoma. Sci Rep. 2024;14:1291. 38221530 10.1038/s41598-024-51794-1 PMC10788337

[29] Subhi-Issa N, Tovar Manzano D, Pereiro Rodriguez A, Sanchez Ramon S, Perez Segura P, Ocana A. Gammadelta T cells: game changers in immune cell therapy for cancer. Cancers. 2025;17. 10.3390/cancers17071063 PMC11987767 40227601

[30] Duval K, Grover H, Han L, Mou Y, Pegoraro AF, Fredberg J, et al. Modeling physiological events in 2D vs. 3D Cell Cult Physiol. 2017;32:266–77. 10.1152/physiol.00036.2016 PMC5545611 28615311

[31] Fontoura JC, Viezzer C, Dos Santos FG, Ligabue RA, Weinlich R, Puga RD, et al. Comparison of 2D and 3D cell culture models for cell growth, gene expression and drug resistance. Mater Sci Eng C-Mater Biol Appl. 2020;107:110264. 31761183 10.1016/j.msec.2019.110264

[32] Ma L, Feng Y, Zhou Z. A close look at current gammadelta T-cell immunotherapy. Front Immunol. 2023;14:1140623. 37063836 10.3389/fimmu.2023.1140623 PMC10102511

[33] Kabelitz D, Serrano R, Kouakanou L, Peters C, Kalyan S. Cancer immunotherapy with gammadelta T cells: many paths ahead of us. Cell Mol Immunol. 2020;17:925–39. 32699351 10.1038/s41423-020-0504-x PMC7609273

[34] Semilietof A, Stefanidis E, Gray-Gaillard E, Pujol J, D’Esposito A, Reichenbach P, et al. Preclinical model for evaluating human TCRs against chimeric syngeneic tumors. J Immunother Cancer. 2024;12. 10.1136/jitc-2024-009504 PMC11667476 39794936

[35] Marshall LJ, Bailey J, Cassotta M, Herrmann K, Pistollato F. Poor translatability of biomedical research using animals - a narrative review. ATLA-Altern Lab Anim. 2023;51:102–35. 10.1177/02611929231157756 36883244

[36] Campaner E, Zannini A, Santorsola M, Bonazza D, Bottin C, Cancila V, et al. Breast cancer organoids model patient-specific response to drug treatment. Cancers. 2020;12. 10.3390/cancers12123869 PMC7770601 33371412

[37] Chen D, Xu L, Xuan M, Chu Q, Xue C. Unveiling the functional roles of patient-derived tumour organoids in assessing the tumour microenvironment and immunotherapy. Clin Transl Med. 2024;14:e1802. 10.1002/ctm2.1802 PMC11381553 39245957

[38] Neal JT, Li X, Zhu J, Giangarra V, Grzeskowiak CL, Ju J, et al. Organoid modeling of the tumor immune microenvironment. Cell. 2018;175:1972–88.e16. 30550791 10.1016/j.cell.2018.11.021 PMC6656687

[39] Boj SF, Hwang CI, Baker LA, Chio II, Engle DD, Corbo V, et al. Organoid models of human and mouse ductal pancreatic cancer. Cell. 2015;160:324–38. 25557080 10.1016/j.cell.2014.12.021 PMC4334572

[40] Pauli C, Hopkins BD, Prandi D, Shaw R, Fedrizzi T, Sboner A, et al. Personalized in vitro and in vivo cancer models to guide precision medicine. Cancer Discov. 2017;7:462–77. 28331002 10.1158/2159-8290.CD-16-1154 PMC5413423

[41] Weeber F, van de Wetering M, Hoogstraat M, Dijkstra KK, Krijgsman O, Kuilman T, et al. Preserved genetic diversity in organoids cultured from biopsies of human colorectal cancer metastases. Proc Natl Acad Sci USA. 2015;112:13308–11. 26460009 10.1073/pnas.1516689112 PMC4629330

[42] Galluzzi L, Smith KN, Liston A, Garg AD. The diversity of CD8(+) T cell dysfunction in cancer and viral infection. Nat Rev Immunol. 2025. 10.1038/s41577-025-01161-6 40216888

[43] Cheng L, Ge M, Lan Z, Ma Z, Chi W, Kuang W, et al. Zoledronate dysregulates fatty acid metabolism in renal tubular epithelial cells to induce nephrotoxicity. Arch Toxicol. 2018;92:469–85. 28871336 10.1007/s00204-017-2048-0 PMC5773652

[44] Drost J, Clevers H. Organoids in cancer research. Nat Rev Cancer. 2018;18:407–18. 29692415 10.1038/s41568-018-0007-6

[45] Sato T, Stange DE, Ferrante M, Vries RG, Van Es JH, Van den Brink S, et al. Long-term expansion of epithelial organoids from human colon, adenoma, adenocarcinoma, and Barrett’s epithelium. Gastroenterology. 2011;141:1762–72. 21889923 10.1053/j.gastro.2011.07.050

[46] Boufea K, González-Huici V, Lindberg M, Symeonides S, Oikonomidou O, Batada NN. Single-cell RNA sequencing of human breast tumour-infiltrating immune cells reveals a γδ T-cell subtype associated with good clinical outcome. Life Sci Alliance. 2020;4:e202000680. 33268347 10.26508/lsa.202000680 PMC7723295

[47] Meng Q, Xie S, Gray GK, Dezfulian MH, Li W, Huang L, et al. Empirical identification and validation of tumor-targeting T cell receptors from circulation using autologous pancreatic tumor organoids. J Immunother Cancer. 2021;9:e003213. 34789550 10.1136/jitc-2021-003213 PMC8601084

[48] Li Z, Ma L, Gao Z, Wang X, Che X, Zhang P, et al. Identification and validation of tumor-specific T cell receptors from tumor infiltrating lymphocytes using tumor organoid co-cultures. Cancer Immunol, Immunother. 2024;73:164. 38954022 10.1007/s00262-024-03749-8 PMC11219989

[49] Meier SL, Satpathy AT, Wells DK. Bystander T cells in cancer immunology and therapy. Nat Cancer. 2022;3:143–55. 35228747 10.1038/s43018-022-00335-8

[50] Yuan L, Ma X, Yang Y, Qu Y, Li X, Zhu X, et al. Phosphoantigens glue butyrophilin 3A1 and 2A1 to activate Vgamma9Vdelta2 T cells. Nature. 2023;621:840–48. 37674084 10.1038/s41586-023-06525-3 PMC10533412

[51] Dijkstra KK, Cattaneo CM, Weeber F, Chalabi M, van de Haar J, Fanchi LF, et al. Generation of tumor-reactive T cells by co-culture of peripheral blood lymphocytes and tumor organoids. Cell. 2018;174:1586–98.e12. 30100188 10.1016/j.cell.2018.07.009 PMC6558289

[52] Duhen T, Duhen R, Montler R, Moses J, Moudgil T, de Miranda NF, et al. Co-expression of CD39 and CD103 identifies tumor-reactive CD8 T cells in human solid tumors. Nat Commun. 2018;9:2724. 30006565 10.1038/s41467-018-05072-0 PMC6045647

[53] Janssen A, Villacorta Hidalgo J, Beringer DX, van Dooremalen S, Fernando F, van Diest E, et al. γδ T-cell receptors derived from breast cancer-infiltrating T lymphocytes mediate antitumor reactivity. Cancer Immunol Res. 2020;8:530–43. 32019779 10.1158/2326-6066.CIR-19-0513

[54] Lien SC, Ly D, Yang SYC, Wang BX, Clouthier DL, St. Paul M, et al. Tumor reactive γδ T cells contribute to a complete response to PD-1 blockade in a Merkel cell carcinoma patient. Nat Commun. 2024;15:1094. 38321065 10.1038/s41467-024-45449-y PMC10848161

[55] Sebestyen Z, Prinz I, Déchanet-Merville J, Silva-Santos B, Kuball J. Translating gammadelta (γδ) T cells and their receptors into cancer cell therapies. Nat Rev Drug Discov. 2020;19:169–84. 10.1038/s41573-019-0038-z. 31492944 10.1038/s41573-019-0038-z

[56] Sandstrom A, Peigné C, Léger A, Crooks JE, Konczak F, Gesnel M, et al. The intracellular B30.2 domain of butyrophilin 3A1 binds phosphoantigens to mediate activation of human Vγ9Vδ2 T cells. Immunity. 2014;40:490–500. 10.1016/j.immuni.2014.03.003. 24703779 10.1016/j.immuni.2014.03.003 PMC4028361

[57] Gao D, Vela I, Sboner A, Iaquinta PJ, Karthaus WR, Gopalan A, et al. Organoid cultures derived from patients with advanced prostate cancer. Cell. 2014;159:176–87. 10.1016/j.cell.2014.08.016. 25201530 10.1016/j.cell.2014.08.016 PMC4237931

