# Supplementary material for: Analysis of the tumor reactivity of autologous TILs and allogeneic γδ T cells via tumor organoid–immune cell coculture
Source: J Transl Med. 2026 Apr 25;24:595. doi: 10.1186/s12967-026-07706-0 (PMC13123169; doi:10.1186/s12967-026-07706-0)
Supplement: Supplementary file 1 — Supplementary Material 1 [file 12967_2026_7706_MOESM1_ESM.docx]

**Supplementary tables and figures**

Supplementary Table 1: Composition of Organoid Culture Medium

| **Component** | **Supplier** | **Catalogue number** | **Final Concentration** |
| --- | --- | --- | --- |
| **Base Medium** |  |  |  |
| Advanced DMEM/F12 | Invitrogen | 12634-010 | 1x |
| **Supplements** |  |  |  |
| B27 supplement | Gibco | 17504-044 | 1x |
| Glutamax 100x | Gibco | 35050061 | 1x |
| HEPES | Solarbio | H1095 | 10 mM |
| **Antibiotics** |  |  |  |
| Penicillin/Streptomycin | Gibco | 15140122 | 1% |
| Primocin | Invivogen | ant-pm-1 | 50 µg/mL |
| **Small Molecules/Inhibitors** |  |  |  |
| Y-27632 (Rock inhibitor) | Abmole | M1817 | 5 µM |
| A83-01 | Tocris | 2939 | 500 nM |
| SB 202190 | Sigma | S7067 | 500 nM |
| **Growth Factors** |  |  |  |
| FGF-7 | OrganRegen | 923-FG7-0100 | 5 ng/mL |
| FGF-10 | OrganRegen | 816-FGF-0100 | 20 ng/mL |
| EGF | Peprotech | AF-100-15 | 5 ng/mL |
| Noggin | Novoprotein | CB89 | 100 ng/mL |
| R-spondin-1 | Novoprotein | CX83 | 250 ng/mL |
| Heregulin β-1 | Peprotech | 100-03-50 | 5 nM |
| **Other Additives** |  |  |  |
| Nicotinamide | Sigma | N0636-100G | 5 mM |
| N-Acetylcysteine | Sigma | A9165-5G | 1.25 mM |

Figures


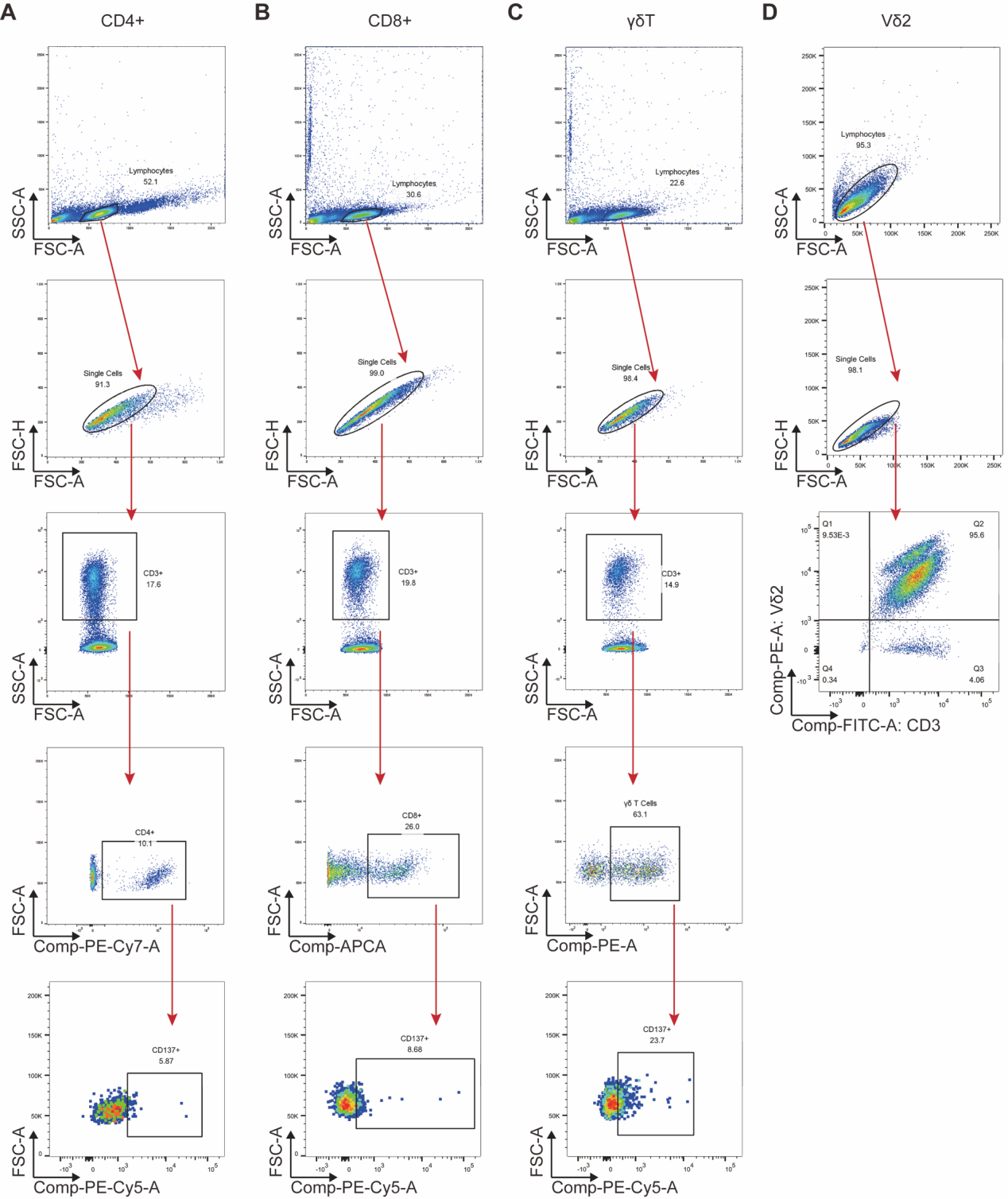


Supplementary Figure 1. Gating strategy for flow cytometric analysis of TIL subsets.

(A) Gating strategy for CD4⁺ T cells. Lymphocytes were first identified based on FSC-A vs. SSC-A gating. Single cells were selected using FSC-H vs. FSC-A to exclude doublets. CD4⁺ T cells were gated within the CD3⁺ population, followed by analysis of CD137 expression as a marker of activation.

(B) Gating strategy for CD8⁺ T cells. Similar to (A), lymphocytes were gated based on FSC-A vs. SSC-A, followed by single-cell selection. CD8⁺ T cells were identified within the CD3⁺ population, and CD137 expression was assessed.

(C) Gating strategy for γδ T cells. Lymphocytes were gated based on FSC-A vs. SSC-A, followed by single-cell selection. γδ T cells were identified within the CD3⁺ population, and CD137 expression was measured.

(D) Gating strategy for Vγ9Vδ2 T cells. Lymphocytes were first gated as in (A-C), followed by single-cell selection. CD3⁺ γδ T cells were further analyzed based on Vγ9Vδ2 expression to distinguish this subset.


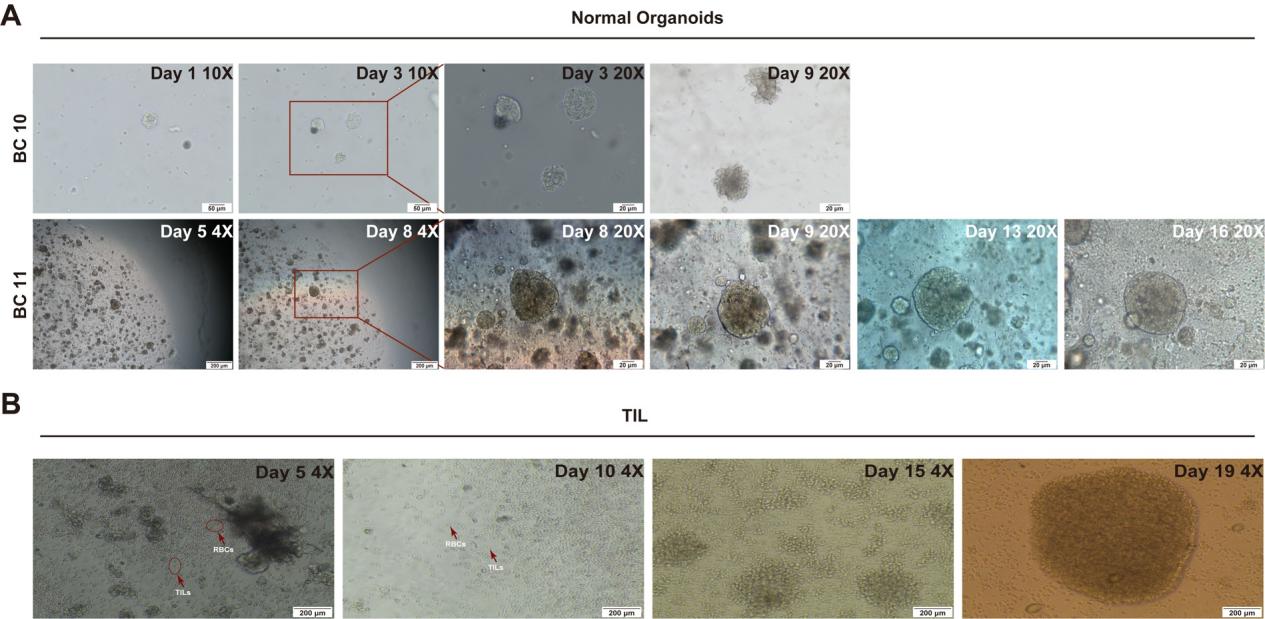


Supplementary Figure 2. Growth of breast cancer organoids and expansion of TILs of breast cancer.

1. Bright-field images of normal breast organoids. the upper panel, the normal organoids from BC10 during 9 day culture, in which the obtained normal epithelial cells are relatively few; the pictures show the processing of organoid formation from cluster cells, slow growth as well as budding; the the down panel, the normal organoids from BC11 from D5-D16 culture, in which the obtained normal epithelial cells are relatively many .Scale bars: 200 μm (4X), 50 μm (20X).

(B) TIL culture from the appropriate 1mm^3^ dissected tumor tissue fragments at Day 5, Day 10, Day 15, and Day 19. TILs move out from tumor tissue fragments, progressively expand in culture, and aggregates from scattered single cells to larger clusters. Scale bars: 200 μm (Day 5, Day 19), 50 μm (Day 10).
